# Towards the Standardization of Artificial Aging Protocols for Dental Composites: Evaluation of Proposed Methods

**DOI:** 10.3390/jfb16020049

**Published:** 2025-02-04

**Authors:** Agata Szczesio-Wlodarczyk, Karolina Kopacz, Katarzyna Ranoszek-Soliwoda, Jerzy Sokolowski, Kinga Bociong

**Affiliations:** 1 University Laboratory of Materials Research, Medical University of Lodz, Pomorska 251, 92-213 Lodz, Poland; 2 “DynamoLab” Academic Laboratory of Movement and Human Physical Performance, Medical University of Lodz, 92-213 Lodz, Poland; 3 Faculty of Medical Sciences, Warsaw Medical Academy, 02-091 Warszawa, Poland; 4 Department of Materials Technology and Chemistry, Faculty of Chemistry, University of Lodz, 90-236 Lodz, Poland; 5 Department of General Dentistry, Medical University of Lodz, 92-213 Lodz, Polandkinga.bociong@umed.lodz.pl (K.B.)

**Keywords:** dentistry, resin, composite, aging protocols, degradation, clinical performance

## Abstract

In restorative dentistry, there are no standardized in vitro accelerated aging methods to evaluate the long-term stability of dental composites. Current research aimed at extending the clinical success of restorations emphasizes the need for post-aging evaluation. This study represents the final stage of assessing three selected aging protocols that utilize a 0.1 M sodium hydroxide solution as the primary agent to accelerate degradation processes. Twelve resin-based composites, categorized into five types, were evaluated for flexural strength (FS), diametral tensile strength (DTS), hardness (HV), and fracture toughness (FT) both before and after aging. The proposed aging methods significantly degraded the mechanical properties of most materials, highlighting the effectiveness of 0.1 M NaOH as a medium for hydrolytic stability testing. Materials with a high filler content (approximately 80 wt.%) were notably prone to degradation, underscoring the importance of optimizing the filler and coupling agent. The findings suggest that incorporating thermocycling into aging protocols may enhance the development and evaluation of innovative dental composites. This work contributes to establishing a foundation for standardized aging protocols, supporting the accurate assessment of composites in vitro.

## 1. Introduction

Though largely preventable, oral diseases remain a significant global health burden, impacting individual and public health, quality of life, and economies [1]. Additionally, poor oral health is one of the main causes of the loss of healthy longevity in older people [2]. Considering that many developed countries are experiencing rapidly aging populations, actions aimed at achieving a high healthy age should become key initiatives [3].

Dental composites have become integral to modern restorative dentistry due to their esthetic advantages and good mechanical properties [4]. Nevertheless, the longevity and durability of these materials remain subjects of ongoing research and clinical concern [5,6]. Dental composites in the oral environment are exposed to both chemical and mechanical degradation processes, with hydrolysis playing a crucial role in this mechanism. A key phenomenon occurring in dental restorations is the absorption of water and other substances from the surrounding environment, resulting in the plasticization, swelling, and softening of the polymer matrix [7]. The aqueous environment can cause oxidation and hydrolysis of the key components of dental composites. Siloxane bonds between the filler and the coupling agent, and ester bonds between the filler and the polymer matrix or those present within the polymer matrix are particularly susceptible to degradation [8]. Hydrolysis can be catalyzed by enzymes produced by bacteria present in the oral cavity. Accelerated degradation of dental composites has also been observed in high alkaline (pH 13.0) or very low (pH < 2.0) acidic environments, due to the increased presence and interactions of hydroxide (OH^-^) and hydrogen (H^+^) ions [9]. Emerging research directions focused on enhancing the longevity of dental restorations explore the application of novel, often complex technologies, such as modifications to the polymer matrix (e.g., click chemistry), advancements in fillers (e.g., bactericidal composite fillers), and the optimization of coupling agents [10]. Furthermore, another line of research is focused on the development of self-healing materials [11].

Dental composites are continuously exposed to the challenging conditions of the oral environment, including fluctuations in temperature, pH, and mechanical stresses [12,13,14]. Therefore, it is imperative to develop standardized methods to predict their long-term performance in vitro accurately. Another indisputable value of such standardization is predicting how significantly the properties of the tested materials will deteriorate.

Artificial aging protocols are essential tools in many industries for simulating the long-term effects of usage environment on products [15]. In dentistry, there is still a lack of consensus on the most representative in vitro aging protocols [16]. This absence of standardization impedes the ability to accurately compare different materials’ performance and confidently predict their clinical longevity. Previous research [17,18], and this study, aim to address this critical gap. Considering that the degradation of dental composites occurs via hydrolysis processes, a suitable medium for in vitro aging should be a solution characterized by elevated hydroxyl ion from, i.e., sodium hydroxide (NaOH) [9,19]. Thermal fluctuations (approximately 20 to 50 times daily [20]) in the oral environment should also be considered. The findings from a previous study [17,18] led to the selection of an aging protocol that involved thermocycles and exposure to a harsh environment inducing accelerated hydrolytic degradation (0.1 M NaOH solution). The repeated heating and cooling cycles during thermocycling caused increased flexibility and swelling of the polymer structure, making it more susceptible to penetration by a corrosive aging agent. Thermocycles were replaced with water aging at higher temperatures (55 °C) to streamline this method. These complex aging protocols aim to replicate extended periods of material exposure in the oral cavity, potentially spanning several years, offering valuable insights into the anticipated clinical performance in laboratory settings.

In the final stage of efforts to contribute to the foundation for standardized aging protocols, twelve commercially available resin-based composites were selected, representing various types of dental materials. The null hypothesis posits that the properties of the tested materials will remain stable following in vitro aging.

## 2. Materials and Methods

Three different artificial aging protocols were selected for this study based on previous research [17,18]. The primary aging factor is a 0.1 M NaOH solution, which, due to its high concentration of hydroxyl ions, serves as an effective agent for accelerating hydrolysis. Two aging protocols include more complex procedures, where thermocycling or aging at elevated temperatures precedes exposure to the degrading medium. These preliminary steps are designed to enhance the flexibility and swelling of the polymer structure, potentially amplifying the material’s response to hydrolytic degradation. A detailed description of chosen aging methods is shown in Table 1.

Twelve various resin composites were used to evaluate the proposed aging protocols. The composition and additional information about the selected materials are presented in Table 2.

The materials were irradiated during sample preparation according to the manufacturer’s recommendations (Table 2) using an LED lamp (The Cure—TC—01, Spring Health Products, Norristown, PA, USA). The lamp’s power was verified with a curing light meter (Light Meter 200, Jovident System, Eindhoven, The Netherlands) and measured to be over 1250 mW/cm^2^.

The effect of the selected aging protocols was evaluated by examining flexural strength (FS), diametral tensile strength (DTS), Vickers hardness (HV), fracture toughness (FT), and microstructure analysis.

### 2.1. Flexural Strength and Modulus

Flexural strength (FS) was assessed using the three-point bending test in accordance with the ISO 4049:2019 standard [22]. The flexural modulus (FM) was determined by the software using the stress–strain curve within the strain range of 0.1% to 0.5%. Seven rectangular samples (2 mm × 2 mm × 25 mm) were prepared per group. A universal testing machine (Z020, Zwick–Roell, Ulm, Germany) was used for the tests.

### 2.2. Diametral Tensile Strength

For the diametral tensile strength (DTS) tests, nine cylindrical samples per group were used with a diameter (*d*) of 6 mm and a height (*h*) of 3 mm. The tests were conducted based on the American Dental Association Specification No. 27 [23]. The test was conducted with 2 mm/min crosshead speed using a universal testing machine (Z020, Zwick–Roell, Ulm, Germany). The *DTS* value based on the maximum force applied (*F*) was calculated using Formula (1).

(1) $$DTS=\frac{2F}{\pi dh}$$

### 2.3. Vickers Hardness

To evaluate hardness, the Vickers method was employed using a Zwick hardness tester (ZHVµm, Zwick–Roell, Ulm, Germany). The measurement load was 1000 g for 10 s. For each experimental group, three randomly selected DTS samples were used, with three measurements taken on each sample, resulting in a total of nine measurements per group.

### 2.4. Fracture Toughness

Six notched rectangular specimens (2 mm × 4 mm × 20 mm) per group were prepared in a metal mold. At the midpoint of the mold, a precisely fabricated slot was utilized to insert a blade, thereby creating a sharp central notch in the sample. Samples were subjected to a three-point bending test in a universal testing machine (Zwick Roell Z005, Ulm, Germany) with 1 mm/min crosshead speed.

The fracture toughness (*FT*) was calculated using Formula (2):

(2) $$FT=\left[\frac{P\cdot S}{B\cdot W^{3/2}}\right]\cdot f\left(\frac{a}{W}\right),$$

where *P* is the peak load at fracture; *S* is the span (14 mm); *B* is the specimen thickness; *W* is the specimen width; *a* is notch length; and $f\left(\frac{a}{w}\right)$ is the function based on formula: $f\left(\frac{a}{w}\right)=2.9{\left[\frac{a}{W}\right]}^{\frac{1}{2}}-4.6{\left[\frac{a}{W}\right]}^{\frac{3}{2}}+21.8{\left[\frac{a}{W}\right]}^{\frac{5}{2}}-37.6{\left[\frac{a}{W}\right]}^{\frac{7}{2}}+38.7{\left[\frac{a}{W}\right]}^{\frac{9}{2}}$ [24].

### 2.5. Water Absorption

Water absorption was determined using five cylindrical samples (15 mm in diameter and 1 mm in height) per dental composite. The samples were cured in nine partially overlapping zones as described in ISO 4049 in sorption/solubility tests [22]. The weight measurements were performed (AS 160/C/2, Radwag, Radom, Poland) immediately after preparation and after 28 consecutive days (4 weeks). According to Formula (3) the absorbency was calculated:

(3) $$A=\frac{m_{i}-m_{0}}{m_{0}}\cdot 100\%,$$

*A*—water absorption,
*m*_0_—the initial mass of the sample,
*m_i_*—the mass of the sample after storage in water for a specified (*i*) period of time.

### 2.6. Microstructure Evaluation

A high-resolution scanning electron microscope (HR-SEM) (FEI Nova NanoSEM 450, FEI, Hillsboro, OR, USA) equipped with a high-sensitivity circular backscatter (CBS) detector was used for a microstructure evaluation of selected samples. Samples were polished and coated with a 10 nm layer of gold (Q15OT ES, Quorum Technologies, Laughton, UK; sputter conditions: material—gold; coating thickness –10 nm; sputter current—40 mA; tooling factor—2.70) before analysis.

### 2.7. Statistical Analysis

Data were analyzed using Statistica version 13 software (StatSoft, Kraków, Poland). The Shapiro–Wilk test was employed to assess the normal distribution of the variables. The data were appropriately analyzed with a significance level set at *p* = 0.05, using the appropriate parametric (ANOVA with a post hoc test (Fisher’s least significant difference)) or non-parametric (Kruskal–Wallis test with multiple comparisons of mean ranks) tests. Additionally, Spearman’s rank order correlations between analyzed research methods were determined.

## 3. Results

### 3.1. Flexural Strength and Modulus

The obtained flexural strengths and modulus of the tested materials are presented in Table 3 and Table 4.

The highest flexural strength under control conditions was observed for Charisma Topaz (148.0 ± 33.0 MPa) and Filtek Bulk Fill Flowable Restorative (121.8 ± 11.0 MPa). A significant strength reduction was observed for most materials, with the smallest decline noted for Revolution Formula 2.

### 3.2. Diametral Tensile Strength

The obtained diametral tensile strengths of the tested materials are presented in Table 5.

In control conditions, Grandio So Heavy Flow had the highest DTS at 63.6 ± 8.0 MPa, while Admira Fusion had the lowest at 35.0 ± 11.2 MPa. Significant changes in DTS values were recorded for seven materials. For the other materials, these changes varied, and for Filtek Bulk Fill Flowable, the values after aging were higher than before.

### 3.3. Vickers Hardness

The obtained hardnesses of the tested materials are presented in Table 6.

The hardness values range from 85 HV (Filtek Ultimate) to 25 HV (Revolution Formula 2). The largest decreases after aging were recorded for Filtek Ultimate, while the smallest was for Revolution Formula 2.

### 3.4. Fracture Toughness

Table 7 presents the tested materials’ fracture toughness.

The material Charisma Topaz (1.92 ± 0.14 MPa√m) exhibited the highest fracture toughness, while the lowest was observed in Admira Fusion (0.88 ± 0.16 MPa√m). The most significant decreases in fracture toughness were noted for the materials Kalore and Neospectra, whereas some of the lowest changes were observed for Revolution Formula 2 and Charisma Bulk Flow One.

### 3.5. Water Absorption

The obtained water absorption after 28 days (4 weeks, 672 h) of the tested materials is presented in Table 8.

**Figure 1:**
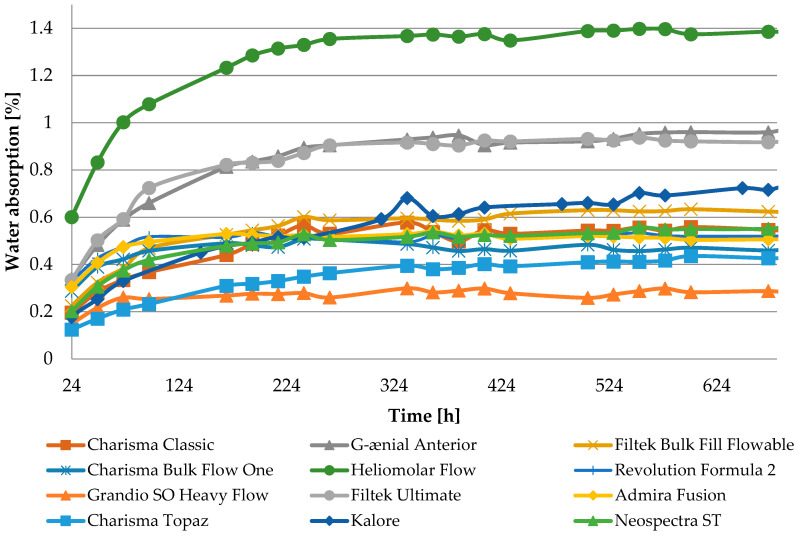
Water absorption curves as a function of time of tested materials.

Water absorbency dynamic plots are presented in Figure 1.

### 3.6. Microstructure Evaluation

**Figure 2:**
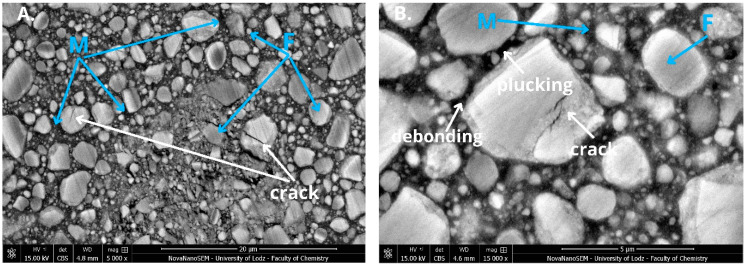
Scanning electron microscopy (SEM) micrograph of Filtek Ultimate at ×5000 (**A**) and ×15,000 magnification (**B**) after aging (water, 7500 thermocycles, 5/55 °C and 0.1 M NaOH 7 days, 60 °C. Filler particles (F), polymer matrix (M), cracks in the filler particles (crack), rinsed-out filler particles (plucking), and degraded bonding areas between the filler particles and the resin matrix (debonding) are marked.

**Figure 3:**
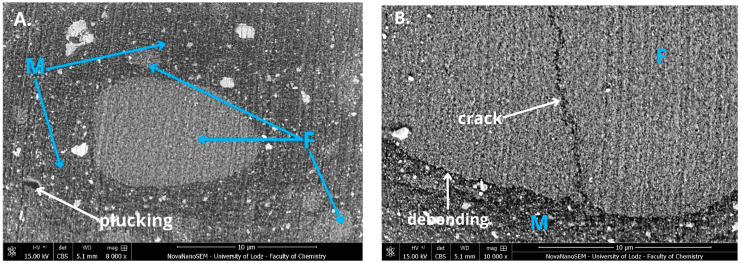
Scanning electron microscopy (SEM) micrograph of Heliomolar Flow at ×5000 (**A**) and ×15,000 magnification (**B**) after aging (water, 7500 thermocycles, 5/55 °C and 0.1 M NaOH, 7 days, 60 °C. Filler particles (F), polymer matrix (M), cracks in the filler particles (crack), rinsed-out filler particles (plucking), and degraded bonding areas between the filler particles and the resin matrix (debonding) are marked.

**Figure A1:**
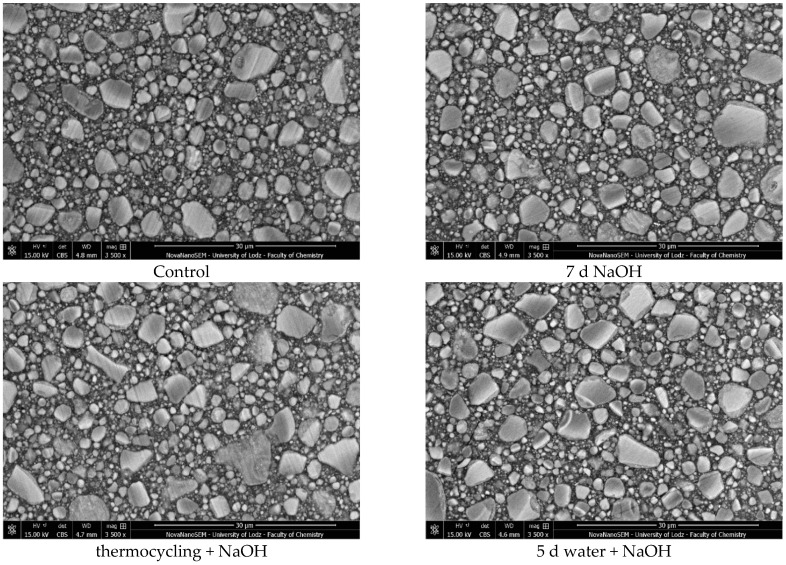
Scanning electron microscopy (SEM) micrographs of Filtek Ultimate at ×3500 magnification after three aging protocols: control—water, 24 h, 37 °C; 7 d NaOH—0.1 M NaOH, 7 days, 60 °C; thermocycling + NaOH—water, 7500 thermocycles, 5/55 °C and 0.1 M NaOH, 7 days, 60 °C; and 5 d water + NaOH—water, 5 days, 55 °C and 0.1 M NaOH, 7 days, 60 °C.

**Figure A2:**
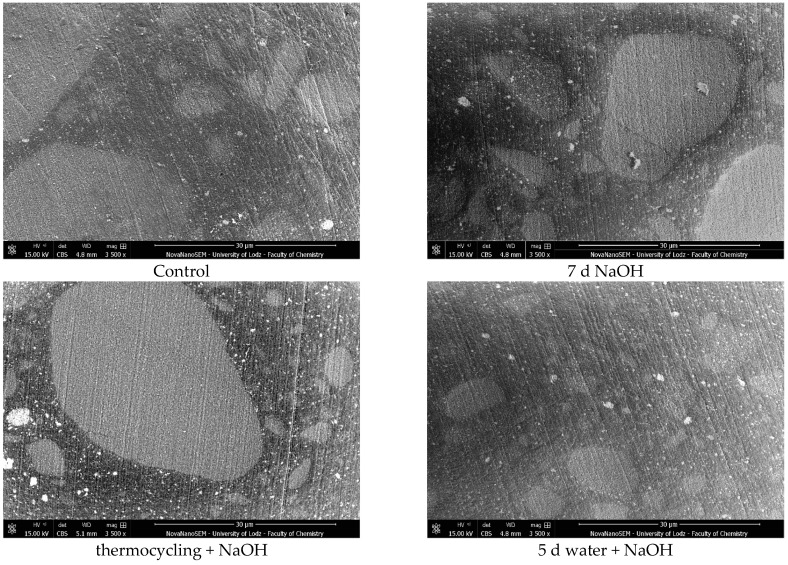
Scanning electron microscopy (SEM) micrographs of Heliomolar Flow at ×3500 magnification after three aging protocols: control—water, 24 h, 37 °C; 7 d NaOH—0.1 M NaOH, 7 days, 60 °C; thermocycling + NaOH—water, 7500 thermocycles, 5/55 °C and 0.1 M NaOH, 7 days, 60 °C; and 5 d water + NaOH—water, 5 days, 55 °C and 0.1 M NaOH, 7 days, 60 °C.

In the SEM micrographs (Figure 2 and Figure 3; Figure A1 and Figure A2) of composite surfaces, both the polymer matrix (M) and filler particles (F) are visible. In the case of Heliomolar Flow, the filler particles exhibit a less homogeneous distribution compared to Charisma Classic. This lack of homogeneity arises from the varying sizes of the filler particles in Heliomolar Flow, where both large and smaller particles are distinctly observable. In contrast, while the filler particles in Charisma Classic also vary in size, the range of variation is lower, contributing to a more uniform distribution. Additionally, Heliomolar Flow exhibits a greater proportion of the polymer matrix compared to Charisma Classic.

The micrographs revealed significant surface deterioration in the dental materials following aging (Figure 2 and Figure 3). Notably, cracks in larger filler particles and instances of rinsed-out filler particles (plucking) were observed in both highly filled and flowable composites. Additionally, degraded bonding areas between filler particles and the resin matrix (debonding) were apparent, indicating compromised interface integrity. These observations underscore the susceptibility of the filler–matrix interface to environmental stresses, which can lead to material degradation over time.

## 4. Discussion

Studies conducted in vitro should provide essential information to accurately assess the material’s suitability under its intended conditions of use. The lack of a standardized aging protocol complicates the evaluation of long-term properties of dental composites under laboratory conditions [16]. This study aimed to assess the impact of various aging protocols on selected dental composites’ mechanical properties, which may contribute to developing a unified method for determining their durability in vitro.

Performing in vitro aging significantly reduced the mechanical properties of most of the materials (Table 2, Table 3 and Table 4, Figure 2 and Figure 3), allowing for the rejection of the stated null hypothesis. Our studies are consistent with other research that showed mechanical properties decrease after artificial aging [19,25,26,27]. Unfortunately, due to the lack of standardized aging methods, accurate comparison of individual studies is challenging. However, the observed changes arise from processes triggered by the exposure of materials to a harsh environment.

Dental composites used for lost tooth tissue restorations are exposed in the oral environment to agents that contribute to their chemical degradation [28]. A primary mechanism is diffusion, wherein water molecules are absorbed by the polymer material, occupying microvoids and free spaces within the polymer matrix. This process causes the plasticization and swelling of the material, with residual monomer particles potentially leaching out and creating additional voids that can further absorb the surrounding medium [7,28,29]. The consequences of diffusion are not only physical—such as plasticization, softening, and swelling of the polymer matrix—but also chemical, as exposure to the aqueous environment leads to the hydrolytic degradation of key bonds in the resin composite, including ester, urethane, amide, and siloxane bonds [30,31]. Although degradation in pure water occurs relatively slowly (depending on time and temperature) [32], it is important to recognize that factors in the oral environment, such as enzymes, temperature fluctuations, chemical substances, and variable pH levels, can accelerate these processes [33].

**Figure 4:**
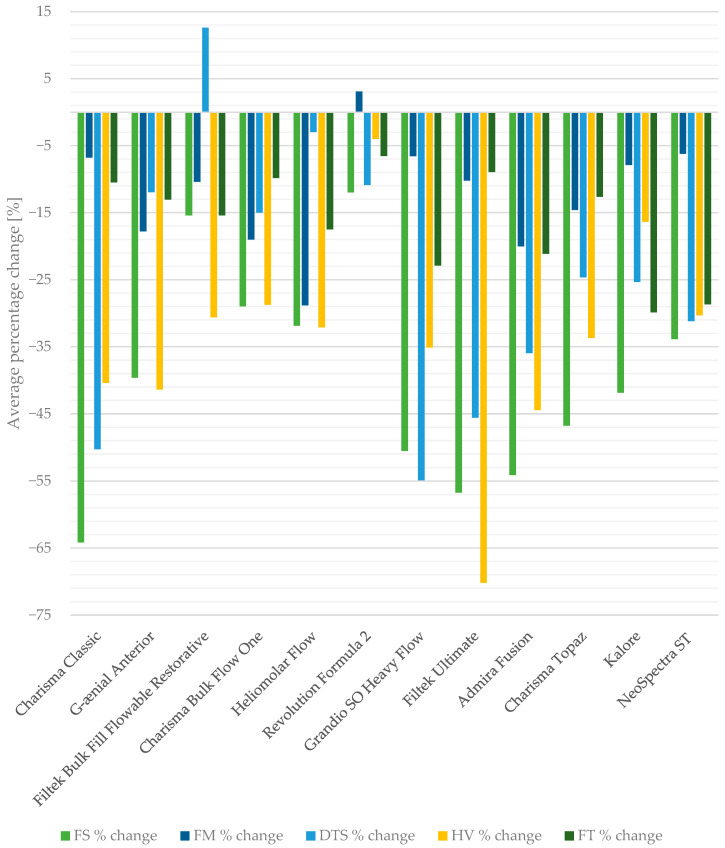
The average percentage changes in the flexural strength (FS), flexural modulus (FM), diametral tensile strength (DTS), Vickers hardness (HV), and fracture toughness (FT) values of the tested materials after the selected aging protocols. Key: 0—no change; positive value—the selected property was higher than the control value after applying aging protocol.

**Figure A3:**
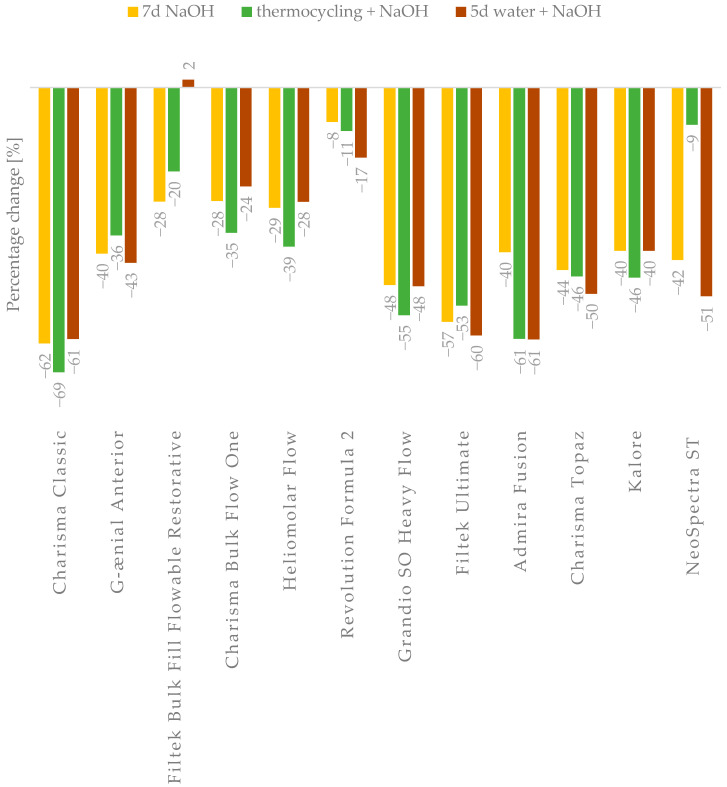
Percentage changes in the flexural strength (FS) values of the tested materials after the selected aging protocols. Key: 0—no change; positive value—the selected property was higher than the control value after applying the protocol. The aging conditions used were as follows: 7 d NaOH—0.1 M NaOH, 7 days, 60 °C; thermocycling + NaOH—water, 7500 thermocycles, 5/55 °C and 0.1 M NaOH, 7 days, 60 °C; 5 d water + NaOH—water, 5 days, 55 °C and 0.1 M NaOH, 7 days, 60 °C.

**Figure A4:**
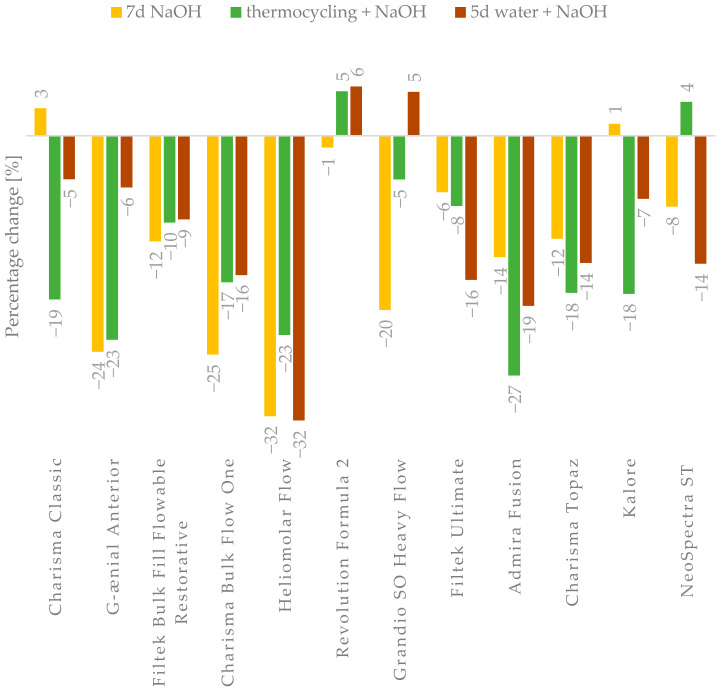
Percentage changes in the flexural modulus (FM) values of the tested materials after the selected aging protocols. Key: 0—no change; positive value—the selected property was higher than the control value after applying the protocol. The aging conditions used were as follows: 7 d NaOH—0.1 M NaOH, 7 days, 60 °C; thermocycling + NaOH—water, 7500 thermocycles, 5/55 °C and 0.1 M NaOH, 7 days, 60 °C; 5 d water + NaOH—water, 5 days, 55 °C and 0.1 M NaOH, 7 days, 60 °C.

**Figure A5:**
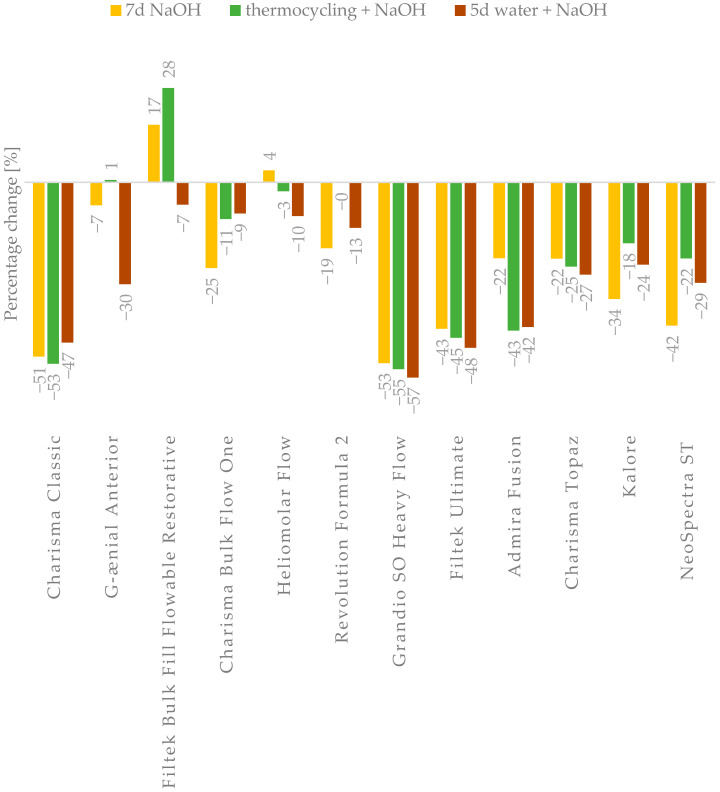
Percentage changes in diametral tensile strength (DTS) values of the tested materials after the selected aging protocols. Key: 0—no change; positive value—the selected property was higher than the control value after applying the protocol. The aging conditions used were as follows: 7 d NaOH—0.1 M NaOH, 7 days, 60 °C; thermocycling + NaOH—water, 7500 thermocycles, 5/55 °C and 0.1 M NaOH, 7 days, 60 °C; 5 d water + NaOH—water, 5 days, 55 °C and 0.1 M NaOH, 7 days, 60 °C.

**Figure A6:**
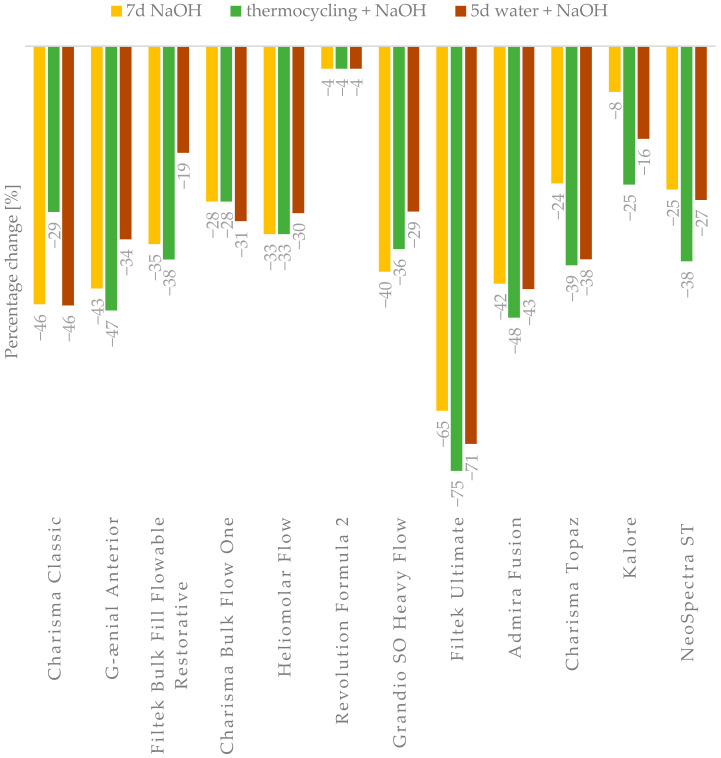
Percentage changes in Vickers hardness (HV) values of the tested materials after the selected aging protocols. Key: 0—no change; positive value—the selected property was higher than the control value after applying the protocol. The aging conditions used were as follows: 7 d NaOH—0.1 M NaOH, 7 days, 60 °C; thermocycling + NaOH—water, 7500 thermocycles, 5/55 °C and 0.1 M NaOH, 7 days, 60 °C; 5 d water + NaOH—water, 5 days, 55 °C and 0.1 M NaOH, 7 days, 60 °C.

**Figure A7:**
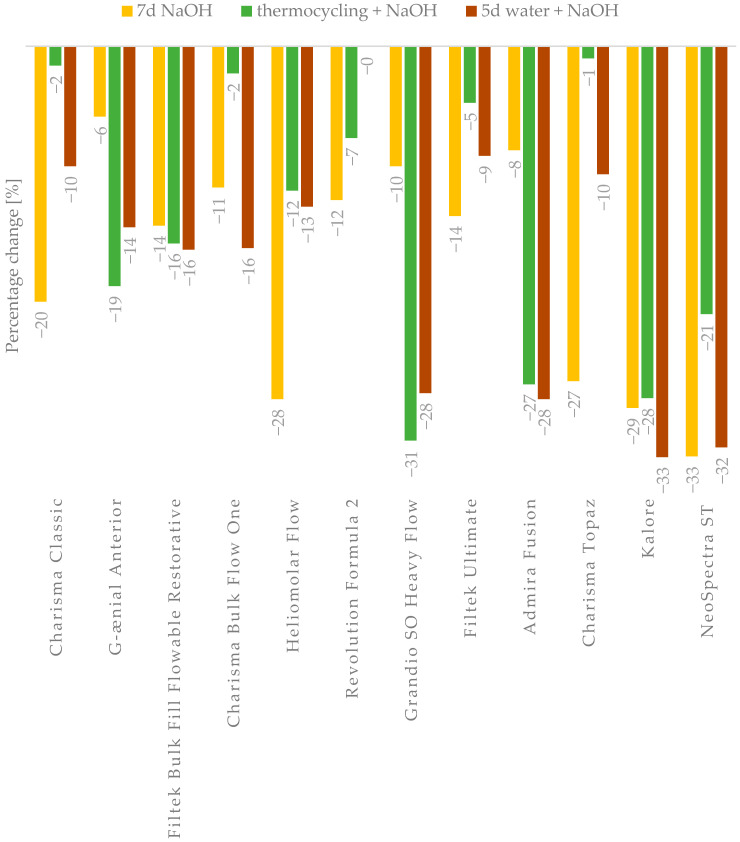
Percentage changes in the fracture toughness (FT) values of the tested materials after the selected aging protocols. Key: 0—no change; positive value—the selected property was higher than the control value after applying the protocol. The aging conditions used were as follows: 7 d NaOH—0.1 M NaOH, 7 days, 60 °C; thermocycling + NaOH—water, 7500 thermocycles, 5/55 °C and 0.1 M NaOH, 7 days, 60 °C; 5 d water + NaOH—water, 5 days, 55 °C and 0.1 M NaOH, 7 days, 60 °C.

In general, aging processes affecting composite materials ultimately reduce their strength properties. However, the obtained results indicate that not all materials experience the same degree of property degradation (Figure 4, Figure A3, Figure A4, Figure A5, Figure A6 and Figure A7).

The study investigated whether these changes correlate with key factors influencing material properties: composition and water sorption. Unfortunately, manufacturers do not provide detailed compositional information, with filler content as the only quantitative data available. It has been shown that mechanical properties such as hardness, flexural strength, and fracture toughness depend both on the amount and type of filler. High filler content, homogeneous particle dispersion, nanofillers’ inclusion, and the filler–matrix interface’s good quality contribute to improved mechanical properties [10,34,35,36,37]. Correlation analysis revealed (Table A1) that mechanical properties, particularly DTS and FS, tend to undergo more significant changes in materials with higher filler content. The authors identified only one study in the literature that compared changes after aging to the amount of organic content in resin composites. In that study [38], no similar correlations were observed. However, the aging protocol involved the use of ethanol as the medium, with observed changes compared to samples stored in water for an equivalent period, rather than to control samples as in the present research. The results revealed that materials with higher filler content demonstrate increased resistance to degradation. However, the study also highlighted the significant impact of coupling agent content on material stability, with higher amounts adversely affecting long-term performance [38]. It is essential to emphasize that increasing the filler content beyond 60% by volume has no measurable impact on enhancing the materials’ mechanical properties [39]. This observation aligns with our findings showing that materials with exceptionally high filler content, such as Grandio, Filtek Ultimate, Admira Fusion, and Kalore, tend to exhibit reduced stability after aging. A dual mechanism can explain the observed behavior. First, the high filler content increases the probability of defects forming within the composite material, compromising its structural integrity [39]. Second, the large interface surface area in such materials, especially those used in nanotechnology, is considered a critical weak point, as reported in the literature. It was shown that exposure to hydroxyl ion-rich solutions, such as 0.1N NaOH, accelerates the hydrolysis of siloxane bonds and filler dissolution. Degradation of this interfacial layer results in the deboning of fillers and rinsing them out, compromising material integrity [9,40,41]. In addition, this process creates new pathways for medium infiltration, further accelerating the deterioration of the material’s performance [30,42]. The selection of an appropriate combination of filler systems and coupling agents presents a significant challenge to the development of new dental materials. Some materials exhibit suboptimal hydrolytic resistance despite technological advancements, as observed in products like Admira Fusion and Kalore. This highlights the need for innovative approaches to improve the stability and durability of composite formulations under an aggressive oral environment.

The observed properties changes do not correlate with water absorption (Table A1). Heliomolar Flow, for example, exhibited the highest water sorption but did not demonstrate the most severe reduction in tested properties. This may be explained by increased material flexibility, which could enhance strength by allowing some plastic deformation during testing. In particular, DTS values may be inflated, as this test is intended for materials that remain brittle and do not undergo plastic deformation under load [43]. A reduction in the modulus of elasticity was observed for most materials (Table 4, Figure A4). The most significant changes were noted for Heliomolar Flow, which may explain the smaller reductions in DTS and FS values after aging, likely due to some degree of material plasticization. Nevertheless, the significant drop in hardness following aging strongly suggests that the material undergoes degradation. Reduced surface hardness by material degradation can lead to increased abrasive wear, which may have adverse clinical implications by compromising the longevity and functionality of the restoration [44,45].

The Grandio SO HF material exhibits the lowest sorption values, likely due to its high filler content exceeding 80 wt.%, compared to 60 wt.% in Revolution Formula 2, despite their similar compositions [46,47]. However, considering overall stability, from our study objects, Kerr’s materials demonstrate superior performance, whereas Voco’s materials are less stable. It is most likely that in this material, the choice of filler and the quality of the filler–matrix interface were the issues. While Grandio SO HF shows lower sorption values, its significant hydrolytic instability is likely due to the high content of glass fillers. Unfortunately, no information is available on the type of coupling agent used, which also plays a key role. As discussed earlier, high filler content may result in worse resistance to hydrolysis due to the presence of internal flaws and a large interface that is susceptible to hydrolysis. Research also indicates that dental fillers can be leached out from the material, especially some glass fillers [26]. Degradation of the filler–matrix interface compromises material integrity, and further debonding processes may result in filler particle leaching. This process creates voids in the composite material, allowing bacterial infiltration, altering the local pH, and accelerating composite hydrolysis during clinical use [9,40,48].

Regarding flexural strength, most materials initially meet the requirements of ISO 4049:2019 (FS > 80 MPa) for composites intended for occlusal use [22], with exceptions observed for Admira Fusion and G-ænial Anterior. In a previous paper [18], there was a proposition that materials should achieve post-accelerated aging flexural strength of 32–48 MPa. The suggested range of limit values was based on the average masticatory forces (8–12 MPa) and the ISO 4049 standard, which require the flexural strength (FS) to exceed 80 MPa—approximately ten times the average chewing forces. In addition, an estimated fatigue strength threshold of approximately 40% of the FS after aging was considered. The material Admira Fusion falls below these proposed thresholds, and traditional, highly filled composites (e.g., Charisma Classic and Geanial Anterior) are near the lower limit. The significant percentage drop in FS before and after aging for Charisma Classic and Admira Fusion suggests that the applied aging methods particularly affected these two materials. Notably, materials of the Bulk Fill and Flow types displayed the least degradation and relatively high FS values post-aging. Admira Fusion is an innovative material utilizing ORMOCER^®^ resin (Pure Silicate Technology) as its matrix. Despite initially promising results in preliminary studies [49], it has demonstrated reduced aging resistance [50,51]. This may be attributed to the low degree of conversion caused by the steric hindrance of the highly functionalized ORMOCER^®^ molecule [52].

**Figure A8:**
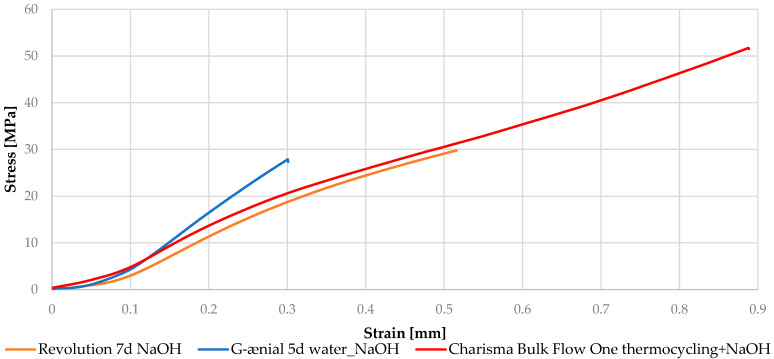
Selected stress–strain curves observed during diametral tensile strength tests.

Diametral tensile strength (DTS), while commonly employed in studies of dental materials, raises concerns regarding the accuracy of its results. The stress–strain curves for most materials reveal certain plastic deformations, especially after aging, suggesting that modern composite materials do not exhibit the typical brittleness associated with traditional materials. Consequently, DTS values may be overestimated, leading to potential misinterpretations of the data. Selected sample strain–stress curves observed during DTS tests are presented in Figure A8. This issue is particularly relevant when evaluating innovative materials and studies incorporating aging processes, which tend to plasticize the polymer matrix. Therefore, we recommend closely examining stress–strain curves and sample fractures, not just the DTS values themselves to assess the influence of plastic deformations on the results accurately.

Higher fracture toughness (FT) values indicate better clinical performance. It measures a material’s ability to resist crack propagation under stress, which may occur in materials due to exposure to cyclic loading, temperature fluctuations, and chemical challenges [53]. Considering typical values in dentistry, enamel ranges from 0.7 to 1.3 MPa√m and dentin 1.7 to 3 MPa√m [54], while composite materials generally show FT values of 1 to approximately 2 MPa√m [55]. Therefore, if composites remain stable and their FT values exceed 1 MPa√m after aging, they are likely capable of withstanding loads encountered in the oral cavity. Conversely, lower FT values suggest a higher likelihood of material failure under stress. The obtained results confirm this hypothesis. The most significant differences in FT were observed for materials such as Grandio, Admira, Kalore, and Neospectra. These differences may be attributed to inadequate integration between the high filler content and the resin matrix. Fracture toughness is a particularly sensitive indicator of defects, making it an effective method for evaluating material performance and stability. Moreover, this parameter provides insights into the quality of the filler–matrix interface and the material’s resistance to defects, making it a vital criterion for assessing the long-term performance of composite materials in restorative dentistry [55,56,57].

Given the complexity of the oral environment, accurately replicating all its variables is impossible. Therefore, a frequently used approach for in vitro aging involves the application of media such as ethanol or NaOH, which accelerate hydrolytic degradation processes affecting the polymer matrix, the coupling agent, and fillers, especially the glass-based ones [9,40,58,59]. Our findings demonstrate that using such media significantly impacts the mechanical properties of composites and can serve as a valuable method for in vitro studies to assess the hydrolytic stability of resin-based composite materials [60,61]. Additionally, thermocycling incorporation is an important aspect of aging studies. Temperature fluctuations during thermocycling can have a significant impact on the behavior of composite restorations. Thermal loads in the oral cavity arise from exposure to varying temperatures, such as hot or cold foods and beverages, creating thermal fatigue in restorative materials under moist conditions. Heat transfer occurs through convection, resulting in thermal boundary layers and uneven temperature distributions, causing thermal stresses due to differing thermal expansion coefficients or non-uniform material properties. In multiphase materials like composites, filler particles expand differently, further inducing internal stresses, especially at the matrix–filler interface. Those processes lead to the deterioration of the internal materials’ integrity. Cyclic temperature changes primarily affect the surface layer, where stress gradients are highest, increasing the risk of degradation. A reliable indicator of ongoing changes could be surface microhardness [62,63].

Drawing on the findings of this study and previous research [17,18], the application of aggressive media, such as NaOH, has proven effective in creating a consistent framework for evaluating the long-term stability of dental composites under in vitro conditions. This approach enables the observation of changes in mechanical properties resulting from the degradation of individual material components. When developing new composites, it is recommended that researchers conduct accelerated aging tests to identify the most durable ingredients and optimal formulations. Additionally, employing thermocycling during aging protocols can reveal interfacial issues between the matrix and filler that might remain undetected using simpler protocols. To provide a comprehensive evaluation of the material’s mechanical performance fracture, toughness testing should also be incorporated. It is worth emphasizing that results from diametral tensile strength (DTS) tests should be interpreted critically, as some plastic deformations can be induced during the test, potentially skewing the outcomes and limiting their reliability.

The findings highlight challenges associated with highly filled materials, which are currently widely promoted in the market. A particularly sensitive aspect is the matrix–filler interface, which significantly influences the material’s long-term performance. Notably, there is a scarcity of foundational studies addressing the behavior of new, proprietary products introduced by dental companies, underscoring the need for further research to evaluate their properties comprehensively.

## 5. Conclusions

Based on the research conducted, and taking into account its limitations, comments and conclusions can be drawn, as follows:

1. Proposed accelerated aging significantly deteriorates the mechanical properties of most dental materials.
2. A 0.1 M NaOH solution can serve as an effective medium for evaluating the hydrolytic stability of dental materials.
3. Complex aging protocols, particularly involving thermocycling, may be valuable in the development of new highly filled composite materials and studies focusing on modifications of the coupling agent.
4. Highly filled materials (approximately 80 wt.%) are susceptible to substantial degradation. Special attention should be given to optimizing the balance between filler content and the coupling agent.
5. Bulk-fill materials exhibit the highest stability among tested materials.

## Figures and Tables

**Table 1:** Description of the selected aging protocol [18].

Aging Protocol	Description
**Control**	24 h, 37 °C, distilled water
**7 d NaOH**	7 days, 60 °C, 0.1 M NaOH
**thermocycling + NaOH**	7500 cycles, 5 °C and 55 °C, water and 7 days, 60 °C, 0.1 M NaOH
**5 d water + NaOH**	5 days, 55 °C, water and 7 days, 60 °C, 0.1 M NaOH

**Table 2:** Description of resin composites used in the study.

Material	Manufacturer	Composition *	Filler Content	Cure Time [s] *	Type
Charisma Classic	Kulzer GmbH, Hanau, Germany	bis-GMA, TEGDMA, DUDMA, 1,12-Dodecanediol dimethacrylate, methacrylic acid, Barium Aluminum Fluoride glass, Potassium feldspar	77 wt.%, 61 vol%	20	Universal composite—high filler content
G-ænial Anterior	GC Dental Product Corporation, Aichi, Japan	UDMA, dimethacrylate co-monomers (Bis-GMA free), silica, fumed silica, pre-polymerized fillers: silica, strontium, lanthanoid fluoride	73 wt.%, 64 vol%	10	Universal composite—high filler content
Filtek Bulk Fill Flowable Restorative	3M Company, St. Paul, MN, USA	DUDMA, Procrylat resin, BIS-PMA, Bis-GMA, Bis-EMA, TEGDMA, zirconia/silica (0.01–3.5 μm), ytterbium trifluoride (0.1–5.0 μm)	64.5 wt.%, 42.5 vol%	20	Bulk fill composite
Charisma Bulk Flow One	Kulzer GmbH, Hanau, Germany,	Bis-EMA, DUDMA, TEGDMA, Barium Aluminum Fluoride glass, Silicate silica, Ytterbium(III) fluoride	65% wt.%, 41 vol%	20	Bulk fill composite
Heliomolar Flow	Ivoclar Vivadent, Schaan, Liechtenstein	Bis-GMA, UDMA, TEGDMA, highly dispersed silica, prepolymer, Ytterbium trifluoride	59 wt.%, 30 vol%	40	Flowablecomposites—low filler content
Revolution Formula 2	Kerr, Scafati, Italy	Bis-GMA, Bis-EMA, TEGDMA, glass filler (0.6 μm)	60 wt.%	10	Flowablecomposites—low filler content
Grandio SO Heavy Flow	Voco GmbH, Cuxhaven, Germany	BisGMA, TEGDMA, BisEMA, Barium Aluminum Boro Silicate glass, fumed silica	83 wt.%	20	Nano-hybrid composite
Filtek Ultimate	3M ESPE St. Paul, MN, USA	Bis-GMA, UDMA, TEGDMA, and Bis-EMA(6), PEGDMA silica (20 nm), zirconia (4 to 11 nm), zirconia/silica cluster	78.5 wt.%, 63.3 vol%	10	Nano-hybrid composite
Admira Fusion	Voco GmbH, Cuxhaven, Germany	ORMOCER^®^ resin (Pure Silicate Technology), silicon dioxide, Barium aluminum borosilicate glass	84 wt.%	20	Innovative composite—new resin technology: ORMOCER
Charisma Topaz	Kulzer GmbH, Hanau, Germany	TCD-DI-HEA, DUDMA, Barium Aluminum Fluoride glass, pre-polymerized fillers, highly discrete nanoparticles (5 nm–5 mm)	59 vol%	20	innovative composite—unique matrix: TCD-DI-HEA
Kalore	GC Dental Product Corporation, Aichi, Japan	Dimethacrylate co-monomers, DX-511 monomer, DUDMA, Prepolymer (incl. 400 nm SrO2 and 100 nm lanthanoid fluoride), Aluminum Fluoride silicate (700 nm), Strontium Barium-glass (700 nm), silicon dioxide (16 nm)	82 wt.%, 69 vol%	10	innovative composite—unique matrix: DX-511
NeoSpectra ST	Dentsply DeTrey GmbH, Konstaz, Germany	Bis-EMA, TEGDMA, blend of spherical, pre-polymerized SphereTEC^®^ fillers, non-agglomerated barium glass, ytterbium fluoride, polysiloxane nanoparticles	78–80 wt.%, 60–62 vol%	10	innovative composite—new filler technology: SphereTEC^®^

* data given by manufacturers, abbreviation: Bis-GMA—Bisphenol A glycerolate dimethacrylate); Bis-EMA—Bisphenol A ethoxylate dimethacrylate; TEGDMA—triethylene glycol dimethacrylate; DUDMA—Diurethane dimethacrylate; BIS-PMA—(1-Methylethylidene)bis(4,1-phenyleneoxy-3,1-propanediyl) bismethacrylate; UDMA—urethane dimethacrylate; TCD-DI-HEA—2-Propenoic acid, (octahydro-4,7-methano-1H-indene-5-diyl)bis(methyleneiminocarbonyloxy-2,1-ethanediyl) ester; DX-511—Crosslink branching monomer with high molecular weight [21].

**Table 3:** The tested materials’ flexural strength (FS) values following the applied aging protocols. For normally distributed variables, data are presented as means with standard deviations (SDs), whereas for non-normally distributed variables, medians with quartile deviations (QDs) are reported. Results within the same material marked with identical letters indicate statistically significant differences (*p* ≤ 0.05).

	Control [MPa]	7 d NaOH [MPa]	Thermocycling + NaOH [MPa]	5 d Water + NaOH [MPa]
Charisma Classic *	109.0 ± 20.0 ^a^	41.2 ± 7.6	33.6 ± 4.8 ^a^	42.4 ± 3.9
G-ænial Anterior	79.4 ± 6.9 ^a,b,c^	47.4 ± 6.4 ^a^	50.9 ± 8.1 ^b^	45.6 ± 3.6 ^c^
Filtek Bulk Fill Flowable Restorative	121.8 ± 11.0 ^a,b^	88.1 ± 5.0 ^a,c^	97.0 ± 7.8 ^b,d^	124.1 ± 7.6 ^c,d^
Charisma Bulk Flow One	113.2 ± 8.3 ^a,b,c^	81.9 ± 8.0 ^a^	73.2 ± 12.0 ^b,d^	86.0 ± 3.4 ^c,d^
Heliomolar Flow	98.0 ± 6.7 ^a,b,c^	69.4 ± 7.8 ^a^	60.1 ± 4.7 ^b,d^	70.8 ± 13.0 ^c,d^
Revolution Formula 2 *	90.0 ± 18.6 ^a^	82.5 ± 10.6	80.5 ± 11.9	74.7 ± 6.5 ^a^
Grandio So Heavy Flow	119.3 ± 11.5 ^a,b,c^	62.2 ± 13.6 ^a^	53.3 ± 11.9 ^b^	61.7 ± 10.0 ^c^
Filtek Ultimate *	120.0 ± 25.3 ^a,b^	51.7 ± 7.2 ^a^	56.4 ± 5.5	47.7 ± 9.4 ^b^
Admira Fusion *	64.5 ± 10.7 ^a,b^	38.7 ± 17.3	23.9 ± 2.4 ^a^	25.0 ± 4.0 ^b^
Charisma Topaz *	148.0 ± 33.0 ^a,b^	82.4 ± 26.1	80.1 ± 12.4 ^a^	73.8 ± 3.9 ^b^
Kalore *	97.0 ± 1.5 ^a,b,c^	58.5 ± 3.3 ^a^	52.2 ± 10.7 ^b^	58.5 ± 4.0 ^c^
Neospectra ST	89.5 ± 8.1 ^a,b,c^	52.0 ± 5.6 ^a,d,e^	81.4 ± 8.8 ^b,d,f^	44.1 ± 4.4 ^c,e,f^

Aging protocols: control—water, 24 h, 37 °C; 7 d NaOH—0.1 M NaOH, 7 days, 60 °C; thermocycling + NaOH—water, 7500 thermocycles, 5/55 °C and 0.1 M NaOH, 7 days, 60 °C; 5 d water + NaOH—water, 5 days, 55 °C and 0.1 M NaOH, 7 days, 60 °C; *—for this material median value with quartile deviation are presented.

**Table 4:** Flexural modulus (FM) values of the tested materials following the applied aging protocols. For normally distributed variables, data are presented as means with standard deviations (SDs), whereas for non-normally distributed variables, medians with quartile deviations (QDs) are reported. Results within the same material marked with identical letters indicate statistically significant differences (*p* ≤ 0.05).

	Control [MPa]	7 d NaOH [MPa]	Thermocycling + NaOH [MPa]	5 d Water + NaOH [MPa]
Charisma Classic *	9350 ± 780 ^a^	9640 ± 2300 ^b^	7620 ± 750 ^a,b^	8890 ± 310
G-ænial Anterior *	5980 ± 690 ^a,b^	4520 ± 660 ^a^	4600 ± 620 ^b^	5630 ± 1070
Filtek Bulk Fill Flowable Restorative *	5610 ± 440 ^a,b^	4940 ± 90 ^a^	5060 ± 400 ^b^	5080 ± 690
Charisma Bulk Flow One *	4890 ± 350 ^a,b^	3680 ± 220 ^a^	4080 ± 190 ^b^	4120 ± 410
Heliomolar Flow *	4130 ± 500 ^a,b^	2820 ± 250 ^a^	3200 ± 140	2800 ± 1250 ^b^
Revolution Formula 2 *	3780 ± 520	3730 ± 410	3970 ± 140	3990 ± 460
Grandio SO Heavy Flow *	10,100 ± 890	8110 ± 2860 ^a^	9600 ± 940	10,600 ± 500 ^a^
Filtek Ultimate	9691 ± 782 ^a,d^	9071 ± 650 ^b^	8921 ± 625 ^c,d^	8111 ± 672 ^a,b,c^
Admira Fusion	6661 ± 412 ^a,b,c^	5747 ± 476 ^a,d^	4856 ± 240 ^b,d,e^	5380 ± 445 ^c,e^
Charisma Topaz *	10,300 ± 1320 ^a^	9100 ± 1720	8470 ± 700 ^a^	8820 ± 1700
Kalore *	7440 ± 350 ^a^	7540 ± 450 ^b,c^	6110 ± 960 ^a,b^	6910 ± 380 ^c^
Neospectra ST *	8360 ± 410 ^a^	7690 ± 670	8680 ± 640 ^b^	7150 ± 1030 ^a,b^

Aging protocols: control—water, 24 h, 37 °C; 7 d NaOH—0.1 M NaOH, 7 days, 60 °C; thermocycling + NaOH—water, 7500 thermocycles, 5/55 °C and 0.1 M NaOH, 7 days, 60 °C; 5 d water + NaOH—water, 5 days, 55 °C and 0.1 M NaOH, 7 days, 60 °C; *—for this material median value with quartile deviation are presented.

**Table 5:** Diametral tensile strength (DTS) values of the tested materials following the applied aging protocols. For normally distributed variables, data are presented as means with standard deviations (SDs), whereas for non-normally distributed variables, medians with quartile deviations (QDs) are reported. Results within the same material marked with identical letters indicate statistically significant differences (*p* ≤ 0.05).

	Control [MPa]	7 d NaOH [MPa]	Thermocycling + NaOH [MPa]	5 d Water + NaOH [MPa]
Charisma Classic	51.6 ± 6.7 ^a,b,c^	25.3 ± 6.4 ^a^	24.2 ± 3.8 ^b^	27.4 ± 4.3 ^c^
G-ænial Anterior *	40.8 ± 21.8 ^a^	38.1 ± 34.9 ^b^	41.1 ± 2.7 ^c^	28.6 ± 6.7 ^a,b,c^
Filtek Bulk Fill Flowable Restorative *	48.4 ± 12.0	56.5 ± 12.2	61.8 ± 13.8 ^a^	45.2 ± 4.7 ^a^
Charisma Bulk Flow One *	59.1 ± 10.5 ^a^	44.3 ± 9.4 ^a^	52.7 ± 5.7	53.7 ± 13.0
Heliomolar Flow *	41.4 ± 3.3	42.9 ± 5.8	40.3 ± 4.1	37.3 ± 14.7
Revolution Formula 2 *	39.0 ± 4.8 ^a^	31.5 ± 13.3 ^a^	39.0 ± 2.9	33.8 ± 6.7
Grandio So Heavy Flow	63.6 ± 8.0 ^a,b,c^	30.0 ± 12.2 ^a^	28.8 ± 8.0 ^b^	27.3 ± 5.4 ^c^
Filtek Ultimate	57.5 ± 10.1 ^a,b,c^	32.8 ± 5.9 ^a^	31.3 ± 6.1 ^b^	29.7 ± 4.8 ^c^
Admira Fusion *	35.0 ± 11.2 ^a,b^	27.2 ± 7.4	19.8 ± 4.8 ^a^	20.2 ± 1.9 ^b^
Charisma Topaz *	63.3 ± 6.6 ^a,b,c^	49.1 ± 11.8 ^a^	47.7 ± 4.6 ^b^	46.2 ± 5.0 ^c^
Kalore	48.7 ± 6.2 ^a,b,c^	32.1 ± 7.2 ^a,d^	40.0 ± 5.1 ^b,d^	36.9 ± 6.1 ^c^
Neospectra ST	48.2 ± 5.7 ^a,b,c^	28.0 ± 5.9 ^a,d^	37.5 ± 8.1 ^b,d^	34.0 ± 6.8 ^c^

Aging protocols: control—water, 24 h, 37 °C; 7 d NaOH—0.1 M NaOH, 7 days, 60 °C; thermocycling + NaOH—water, 7500 thermocycles, 5/55 °C and 0.1 M NaOH, 7 days, 60 °C; 5 d water + NaOH—water, 5 days, 55 °C and 0.1 M NaOH, 7 days, 60 °C; *—for this material median value with quartile deviation are presented.

**Table 6:** Vickers hardness (HV) values of the tested materials following the applied aging protocols. For normally distributed variables, data are presented as means with standard deviations (SDs), whereas for non-normally distributed variables, medians with quartile deviations (QDs) are reported. Results within the same material marked with identical letters indicate statistically significant differences (*p* ≤ 0.05).

	Control	7 d NaOH	Thermocycling + NaOH	5 d Water + NaOH
Charisma Classic	60 ± 3 ^a,b,c^	32 ± 5 ^a,d^	42 ± 5 ^b,d,e^	32 ± 5 ^c,e^
G-ænial Anterior	40 ± 1 ^a,b,c^	23 ± 2 ^a,d^	21 ± 2 ^b,e^	26 ± 2 ^c,d,e^
Filtek Bulk Fill Flowable Restorative *	37 ± 1 ^a,b^	24 ± 1 ^a^	23 ± 3 ^b,c^	30 ± 1 ^c^
Charisma Bulk Flow One *	29 ± 1 ^a,b,c^	21 ± 3 ^a^	21 ± 5 ^b^	20 ± 1 ^c^
Heliomolar Flow *	27 ± 1 ^a,b^	18 ± 1 ^a^	18 ± 2 ^b^	19 ± 0
Revolution Formula 2 *	25 ± 4	24 ± 0	24 ± 1	24 ± 1
Grandio So Heavy Flow *	75 ± 2 ^a,b,c^	45 ± 19 ^a^	48 ± 14 ^b^	53 ± 12 ^c^
Filtek Ultimate *	85 ± 2 ^a,b^	30 ± 4	21 ± 2 ^a^	25 ± 9 ^b^
Admira Fusion	57 ± 5 ^a,b,c^	33 ± 3 ^a^	30 ± 4 ^b^	33 ± 5 ^c^
Charisma Topaz	63 ± 2 ^a,b,c^	48 ± 4 ^a,d,e^	39 ± 2 ^b,d^	39 ± 3 ^c,e^
Kalore	43 ± 1 ^a,b,c^	39 ± 3 ^a,d,e^	32 ± 4 ^b,d,f^	36 ± 4 ^c,e,f^
Neospectra ST *	55 ± 1 ^a,b,c^	41 ± 8 ^a^	34 ± 3 ^b^	40 ± 7 ^c^

Aging protocols: control—water, 24 h, 37 °C; 7 d NaOH—0.1 M NaOH, 7 days, 60 °C; thermocycling + NaOH—water, 7500 thermocycles, 5/55 °C and 0.1 M NaOH, 7 days, 60 °C; 5 d water + NaOH—water, 5 days, 55 °C and 0.1 M NaOH, 7 days, 60 °C; *—for this material median value with quartile deviation are presented.

**Table 7:** Fracture toughness (FT) values of the tested materials following the applied aging protocols. For normally distributed variables, data are presented as means with standard deviations (SDs), whereas for non-normally distributed variables, medians with quartile deviations (QDs) are reported. Results within the same material marked with identical letters indicate statistically significant differences (*p* ≤ 0.05).

	Control [MPa√m]	7 d NaOH [MPa√m]	Thermocycling + NaOH [MPa√m]	5 d Water + NaOH [MPa√m]
Charisma Classic	1.02 ± 0.10 ^a^	0.81 ± 0.10 ^a,b,c^	1.00 ± 0.06 ^b^	0.92 ± 0.05 ^c^
G-ænial Anterior	1.13 ± 0.17 ^a^	1.06 ± 0.10	0.91 ± 0.14 ^a^	0.96 ± 0.14
Filtek Bulk Fill Flowable Restorative *	1.44 ± 0.16 ^a^	1.23 ± 0.19	1.21 ± 0.05	1.20 ± 0.14 ^a^
Charisma Bulk Flow One	1.37 ± 0.15 ^a,b^	1.21 ± 0.12 ^a^	1.34 ± 0.10 ^c^	1.15 ± 0.08 ^b,c^
Heliomolar Flow *	1.19 ± 0.35	0.85 ± 0.27	1.05 ± 0.06	1.04 ± 0.32
Revolution Formula 2	1.02 ± 0.13	0.89 ± 0.14	0.94 ± 0.07	1.02 ± 0.08
Grandio SO Heavy Flow *	1.16 ± 0.32 ^a^	1.05 ± 0.38	0.79 ± 0.13 ^a^	0.84 ± 0.07
Filtek Ultimate	1.28 ± 0.10 ^a^	1.11 ± 0.11 ^a^	1.22 ± 0.10	1.17 ± 0.19
Admira Fusion *	0.88 ± 0.16 ^a^	0.81 ± 0.12	0.64 ± 0.11	0.63 ± 0.15 ^a^
Charisma Topaz	1.92 ± 0.14 ^a,d^	1.41 ± 0.08 ^a,b,c^	1.91 ± 0.17 ^b,e^	1.73 ± 0.07 ^c,d,e^
Kalore	1.34 ± 0.15 ^a,b,c^	0.95 ± 0.06 ^a^	0.96 ± 0.05 ^b^	0.90 ± 0.08 ^c^
Neospectra ST *	1.30 ± 0.07 ^a,b,c^	0.87 ± 0.08 ^a,d^	1.03 ± 0.07 ^b,d,e^	0.90 ± 0.06 ^c,e^

Aging protocols: control—water, 24 h, 37 °C; 7 d NaOH—0.1 M NaOH, 7 days, 60 °C; thermocycling + NaOH—water, 7500 thermocycles, 5/55 °C and 0.1 M NaOH, 7 days, 60 °C; 5 d water + NaOH—water, 5 days, 55 °C and 0.1 M NaOH, 7 days, 60 °C; *—for this material median value with quartile deviation are presented.

**Table 8:** The absortion values after 28 days in water for the tested materials.

Material	Absorption After 28 Days [wt.%]	
	Mean	SD
Charisma Classic	0.55	0.01
G-ænial Anterior	0.96	0.06
Filtek Bulk Fill Flowable Restorative	0.62	0.02
Charisma Bulk Flow	0.46	0.02
Heliomolar Flow	1.39	0.10
Revolution Formula 2	0.52	0.06
Grandio SO Heavy Flow	0.29	0.03
Filtek Ultimate	0.92	0.03
Admira Fusion	0.51	0.02
Charisma Topaz	0.43	0.01
Kalore	0.72	0.11
Neospectra ST	0.55	0.06

## Data Availability

Data are available in a publicly accessible repository, Zenodo at https://doi.org/10.5281/zenodo.14576242 (accessed on 3 February 2025).

## Abbreviations

The following abbreviations are used in this manuscript:

FS	flexural strength
DTS	diametral tensile strength
HV	Vickers hardness
FT	fracture toughness

## Appendix A

**Table A1:** The Spearman correlations data of analyzed results.

Analyzed Methods	Scatter Plot	Spearman Correlation Coefficient	*p*-Value
Filler content [wt.%] vs. Average percentage change flexural strength (FS) [%]		−0.55342	0.06195
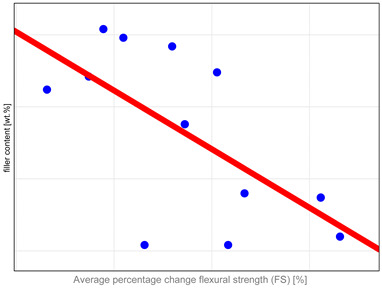
Filler content [wt.%] vs. Average percentage change flexural modulus (FM) [%]		0.231174	0.469732
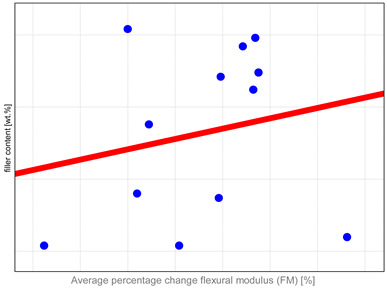
Filler content [wt.%] vs. Average percentage change in diametral tensile strength (DTS) [%]		−0.74256	0.00567
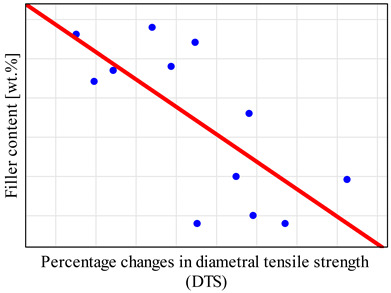
Filler content [wt.%] vs. Average percentage change fracture toughness (FT) [%]		−0.52889	0.07705
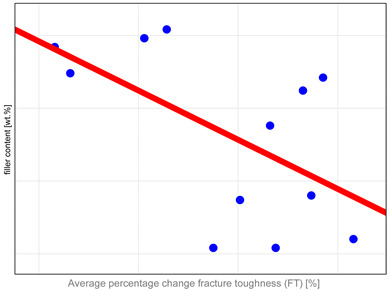
Absorption after 672 h [wt.%] vs. Average percentage change flexural strength (FS) [%]		0.10158	0.753434
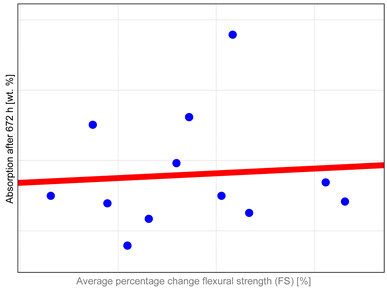
Absorption after 672 h [wt.%] vs. Average percentage change flexural modulus (FM) [%]		−0.23818	0.45599
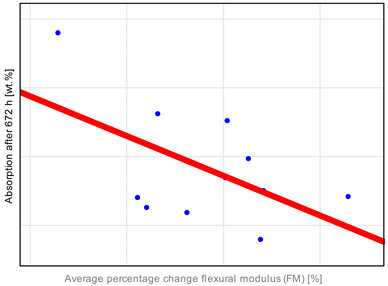
Absorption after 672 h [wt.%] vs. Average percentage change in diametral tensile strength (DTS) [%]		0.38179	0.22071
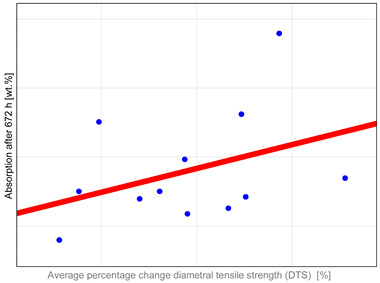
Absorption after 672 h [wt.%] vs. Average percentage change fracture toughness (FT) [%]		−0.04553	0.88825
